# Injection of Anti-proBDNF Attenuates Hippocampal-Dependent Learning and Memory Dysfunction in Mice With Sepsis-Associated Encephalopathy

**DOI:** 10.3389/fnins.2021.665757

**Published:** 2021-07-20

**Authors:** Yan-Hui Cui, Shi-Fen Zhou, Yu Liu, Shuang Wang, Fang Li, Ru-Ping Dai, Zhao-Lan Hu, Chang-Qi Li

**Affiliations:** ^1^Department of Anatomy and Neurobiology, School of Basic Medical Science, Central South University, Changsha, China; ^2^Department of Respiratory and Critical Care Medicine, Xiangya Hospital, Central South University, Changsha, China; ^3^Department of Anesthesiology, The Second Xiangya Hospital, Central South University, Changsha, China; ^4^Department of Medical Research Center and Clinical Laboratory, Xiangya Hospital, Central South University, Changsha, China

**Keywords:** sepsis associated encephalopathy, proBDNF, p75^*NTR*^, hippocampus, cognition and memory dysfunction

## Abstract

Sepsis-associated encephalopathy (SAE) is a risk factor for cognitive and memory dysfunction; however, the mechanism remains unclear. Brain-derived neurotrophic factor (BDNF) was reported to have a positive effect on cognition and emotion regulation, but the study of its precursor, proBDNF, has been limited. This study aimed to elucidate the effects and associated mechanisms of hippocampal proBDNF in a lipopolysaccharide (LPS)-induced SAE mouse model. In this study, we found that the mice exhibited cognitive dysfunction on day 7 after LPS injection. The expression of proBDNF and its receptor, p75^*NTR*^, was also increased in the hippocampus, while the levels of BDNF and its receptor, TrkB, were decreased. A co-localization study showed that proBDNF and p75^*NTR*^ were mainly co-localized with neurons. Furthermore, LPS treatment reduced the expression of NeuN, Nissl bodies, GluR4, NR1, NR2A, and NR2B in the hippocampus of SAE mice. Furthermore, an intrahippocampal or intraperitoneal injection of anti-proBDNF antibody was able to ameliorate LPS-induced cognitive dysfunction and restore the expression of NeuN, Nissl bodies, GluR4, NR1, NR2A, NR2B, and PSD95. These results indicated that treatment with brain delivery by an intrahippocampal and systemic injection of mAb-proBDNF may represent a potential therapeutic strategy for treating patients with SAE.

## Introduction

Sepsis-associated encephalopathy (SAE) is defined as a response of the central nervous system (CNS) to a systemic inflammatory response syndrome and diffuse brain dysfunction (Iwashyna et al., 2010). SAE is associated with mortality in patients with sepsis in the form of delirium, epileptic seizure, and shock (Cohen et al., 2015). A recent study indicated that SAE might induce sustained brain lesions (Semmler et al., 2013). About 40% of patients displayed long-term and irreversible sequela, including memory impairment, depression, anxiety, and cognitive disturbances (Widmann and Heneka, 2014). Thus, the prevention and treatment of SAE, especially the cognitive impairment, is crucial for patients with sepsis.

A clinical study shows that the CNS is indirectly infected during the development of SAE (Gofton and Young, 2012) and that the pathogenesis of SAE involves multiple brain regions (Widmann and Heneka, 2014). The hippocampus, as the primary region of the brain responsible for learning and memory, is sensitive to ischemia, anoxia, and inflammation (Semmler et al., 2005; Hagena et al., 2016). Sepsis survivors were reported to have significant hippocampus atrophy (Semmler et al., 2013). SAE animal models are impaired in hippocampal-dependent learning and memory tasks (Abramova et al., 2013; Chugh et al., 2013). SAE-induced cognitive impairment is related to the dysfunction of neurons and synaptic plasticity of the hippocampus (Christian et al., 2014; Valero et al., 2014). The basis of learning and memory is the modulation of synaptic proteins which influence synaptogenesis and dendritic spine formation that regulate synaptic plasticity (Kariolis et al., 2020). Thus, understanding the mechanisms leading to synaptic and neuronal dysfunction will help to develop the tailored neuronal synapse-targeted therapies for cognitive impairment in SAE.

A reduction in the levels of neurotrophic factors, such as brain-derived neurotrophic factor (BDNF), has been reported in connection with neurogenic defects (Shirayama et al., 2002; Angelucci et al., 2005). BDNF is one of the most abundant and widely distributed neurotrophic factors, which is a major protector of the CNS. BDNF supports neuronal survival, development, and differentiation to regulate learning and memory (Chao, 2003; Reichardt, 2006; Hempstead, 2015). Additionally, BDNF is a critical regulator of synaptic plasticity in the hippocampus (Thoenen, 2000). Previous studies have reported that BDNF protects the CNS by inhibiting apoptosis and impairing neuronal excitation (McAllister, 2002; Counts and Mufson, 2010). Reduced BDNF expression maintains the dysfunction of the CNS in patients with severe sepsis (Ritter et al., 2012).

The precursor form of BDNF, proBDNF, is released extracellularly and can bind its receptor to play an opposing role to mature BDNF (mBDNF) (Koshimizu et al., 2010). proBDNF plays a key role in regulating the development of the CNS and the neurogenesis of the hippocampus *via* binding its high-affinity receptor, p75^*NTR*^, or sortilin (Teng et al., 2005). Studies found that the proBDNF/p75^*NTR*^/sortilin complex stimulated neuronal apoptosis, amyloid deposition, depression, and learning and memory dysfunction in neurodegenerative disease models (Sun et al., 2012; Chen et al., 2016) and that proBDNF levels increased in the brain of patients with Alzheimer’s disease (Chen et al., 2017). Additionally, proBDNF negatively regulates the migration of cerebellar granule cells, and this effect is mediated by p75^*NTR*^ during development and pathological conditions (Xu et al., 2011). Similarly, proBDNF has been reported to inhibit neuronal proliferation and neurogenesis (Li et al., 2017), which may be due, in part, to the activation of RhoA through the p75^*NTR*^ signaling pathway that damages neurite outgrowth and filapodial growth cones *in vitro* (Sun et al., 2012). Furthermore, in a study of the mechanism of pain, proBDNF and p75^*NTR*^ were upregulated in the inflammatory cells of local tissues with inflammatory pain, which was alleviated upon injection of an anti-proBDNF antibody (Luo et al., 2016). Our previous study established that an intraperitoneal injection (i.p.) of lipopolysaccharide (LPS) (20 mg/kg) induced the upregulation of proBDNF in T cells of the mesenteric lymph node (Wang et al., 2019). Furthermore, our recent study demonstrated that the upregulated proBDNF in the immune system promoted the pathogenesis of SAE through downregulating the circulating levels of CD4^+^ T cells, thus limiting its infiltration into the meninges and perturbing the meningeal pro-/anti-inflammatory homeostasis (Luo et al., 2020). Hence, it is plausible that proBDNF modulates the functions of the hippocampus and is involved in regulating the cognitive impairment of SAE.

Here we report that proBDNF and its receptor p75^*NTR*^ were upregulated in hippocampal neurons after the induction of SAE. The systemic administration or intrahippocampal microinjection of anti-proBDNF antibodies (mAb-proB) attenuated cognitive impairment, which may be due to the expansion of neuronal function and the synaptic transmission-associated protein level of the hippocampus in SAE progression. Thus, mAb-proB is a promising therapeutic with the potential to alleviate SAE by regulating the hippocampal neuron function.

## Materials and Methods

### Animals

Male 7- to 8-week-old C57BL/6 mice (18–23 g) were purchased from the Laboratory Animal Co., Ltd., of Slack King (Longping High-Tech Park, Changsha, China) SCXK (Hunan) (2013-0004). The animals were housed at four to five individuals per cage, with a 12-h light/dark cycle at a constant temperature (22°C) and in a humidity-controlled (50 ± 5%) animal facility, with food and water *ad libitum*. All the animal experiments were conducted in accordance with the NIH Guide for the Care and Use of Laboratory Animals (NIH Publications No. 80-23), revised 1996, and were approved by the Animal Ethics Committee of the Third Xiangya Hospital (Changsha, China). All efforts were made to minimize suffering and the number of mice used.

### SAE Mice Model

Lipopolysaccharide derived from *Escherichia coli* serotype 055:B5 (catalog no. L2880, Sigma-Aldrich, United States) was dissolved in 0.9% normal saline and administrated to the mice *via* intraperitoneal injection (i.p. 10 mg/kg) to induce SAE (Zhu et al., 2019; Luo et al., 2020). The control animals were injected with an equivalent volume of saline. The animals were randomly divided into a control group and an LPS group.

### Systemic Administration of Anti-proBDNF Monoclonal Antibody

The neutralizing mAb-proB was developed by Shanghai Yile Biotechnology Company, and its biological function has been well characterized by our previous studies (Luo et al., 2016; Wang et al., 2019; Luo et al., 2020). To investigate the role of mAb-proB, the mice were treated with 100 μg mAb-proB in 0.3 ml normal saline *via* i.p. injection 12 h after the induction of SAE. In the control group, an equal volume of normal mouse IgG isotype (catalog no. AT1596, CMCTAG, United States) was administrated to the mice *via* i.p. injection.

### Intrahippocampal Microinjection of Anti-proBDNF Monoclonal Antibody

MAb-proB was dissolved in saline to a concentration of 1.00 μg/μL. After anesthetization by sevoflurane inhalation and pentobarbital sodium (50 mg/kg), the mice were placed in a stereotaxic apparatus and administered with mAb-proB or normal mouse control IgG (1.00 μg) *via* intrahippocampal injection (AP, 2.06 mm; ML, ±2.30 mm; SV, 2.25 mm relative to the bregma) as previously described (Luo et al., 2020). Bilateral hippocampus infusion was administered *via* 4.20 μl Nanoliter Microinjection with a glass micropipette. Approximately 1.00 μl of mAb-proB or IgG was slowly infused at a rate of 100 nL/s. After an additional 10 min to ensure adequate diffusion, the glass micropipette was slowly retracted from the mouse. At 2 h after the intrahippocampal injection, the mice were administrated with LPS or saline *via* i.p. injection, and their behavior was assessed at 5–9 days after LPS treatment.

### Behavioral and Cognitive Tests

All behavioral procedures were implemented from 9 a.m. to 5 p.m. in a sound-isolated room. Tests were operated and recorded by the same experimenter who was blinded to the grouping of the mice.

#### Open Field Test

The open field is composed of a white polyester resin chamber (50 × 50 × 50 cm^3^). Each mouse was placed in the center of the arena and was free to explore for 5 min. The total distance traveled and the time spent in the central square were recorded and analyzed by the ViewPoint Video Tracking Software (ViewPoint Behavior Technology, Lyon, France).

#### Novel Object Recognition Test

The novel object recognition (NOR) test was carried out in an open field box (50 × 50 × 50 cm^3^). Before the test, the mice were habituated to the box for 5 min without any objects. Then, each mouse was placed in the center of the box and exposed to two identical objects for 5 min (familiarization session) and then returned to their cage. A 30-min interval between the familiarization and test session was set for short-term memory tasks, and a 24-h interval was set for long-term memory tasks. Each mouse was then permitted to explore both the familiar object and a completely different object (novel object) for 5 min (test session). The time spent exploring the familiar object (TA) and the novel object (TB) were recorded and analyzed by the ViewPoint Video Tracking Software (ViewPoint Behavior Technology, Lyon, France). A recognition index, defined as the amount of time spent in exploring the novel object divided by the total time spent in exploring both objects and multiplied by 100 {[TB/(TA + TB)] × 100}, was used to measure recognition memory (Lin et al., 2018).

#### Y Maze Test

The Y maze apparatus was made up of three divided gray polyvinylidene passages, with a 120° angle between each arm (30 × 7 × 16 cm^3^). The apparatus was placed in a soundproof and isolated room. The three arms include the start arm, in which the mouse explored first (always open), the novel arm, which is blocked at the training time but opened at the test time, and the other arm (always open). The Y maze test included two trials–training and testing–separated by an interval time. In the training, each mouse was permitted to explore the start arm and the other arm randomly to avoid spatial memory errors. After a 30-min interval time, each mouse was free to explore all three arms. After a 24-h inter-trial interval, the tags attached to two of these arms were altered, and the mice were allowed to explore the three arms again. The time spent in and the number of entries into each arm were recorded and analyzed *via* the ViewPoint Video Tracking Software (ViewPoint Behavior Technology, Lyon, France).

### Western Blot Analysis

Total protein was extracted from mouse hippocampal tissue using the Minute^TM^ total protein extraction kit (catalog no. SD-001, Invent Biotechnologies Inc., United States) according to the manufacturer’s instructions. Proteins (40 μg) were separated on a 10% SDS-PAGE gel and transferred onto polyvinylidene fluoride membranes (catalog no. IPVH15150, Millipore, Billerica, MA, United States). After blocking with 1% gelatin in Tris-buffered saline plus Tween-20 (TBST) for 1 h at room temperature, the membranes were incubated overnight at 4°C with the following primary antibodies: BDNF antibody, 1:1,000, catalog no. ab108319, Abcam; p75 antibody, 1:1,000, catalog no. ab8874, Abcam; sortilin antibody, 1:2,000, catalog no. ab16640, Abcam; tropomyosin receptor kinase B (TrkB) antibody, 1:1,000, catalog no. 13129-1-AP, Proteintech; β-actin antibody, 1:5,000, catalog no. 66009-1-Ig, Proteintech; GluR1 antibody, 1:1,000, catalog no. ab183797, Abcam; GluR4 antibody, 1:1,000, catalog no. SAB4501296, Sigma; NR1 antibody, 1:1,000, catalog no. ab174309, Abcam; NR2A antibody, 1:1,000, catalog no. 19953-1-AP, Proteintech; NR2B antibody, 1:1,000, catalog no. ab254356, Abcam; and PSD95 antibody, 1:1,000, catalog no. 2507, Cell Signaling Technology. The membranes were rinsed completely and incubated with horseradish peroxidase (HRP)-conjugated goat anti-rabbit or goat anti-mouse secondary antibody (1:2,000, catalog nos. ab6721 and ab6789, Abcam) for 2 h at room temperature. Lastly, the membranes were rinsed and exposed to photographic film with Immobilon western chemiluminescent HRP substrate (catalog no. WBKLS0500, Millipore, United States). The signals were quantified by NIH Image J 7.0 software and standardized to β-actin.

### Immunofluorescence Staining

Paraffin-embedded brain tissues were serially cut into 4-μm sections. The slides were regularly deparaffinized and hydrated. The sections were permeabilized with 0.5% Triton X-100 in phosphate-buffered saline (PBS) for 20 min and blocked with 5% bovine serum albumin (BSA) in Tris-buffered saline for 60 min at 37°C and incubated with the following primary antibodies overnight at 4°C: anti-proBDNF antibody, catalog no. ANT006, 1:500, Alomone lab; anti-NeuN antibody, 1:1,000, catalog no. ab104224, Abcam; and anti-p75NTR antibody, 1:500; catalog no. ab25958, Abcam. The sections were incubated with the following secondary antibodies for 1 h at 37°C [goat polyclonal secondary antibody to rabbit IgG (Goat Anti-Rabbit IgG H&L; Alexa Fluor^®^ 488), 1:1,000 for proBDNF and p75NTR, ab150077, Abcam; goat anti-mouse IgG H&L (Alexa Fluor^®^ 594), 1:1,000 for NeuN]. The coverslips were stained with DAPI (1:2,000, SC-3598, Santa Cruz Biotechnology, Inc., Dallas, TX, United States) for 2 min at room temperature. Immunofluorescence images were acquired using a fluorescence microscope (Nikon ECLIPSE 80i, Nikon Corporation, Tokyo, Japan).

### Nissl Staining

The sections were transferred onto gelatin-coated slides for airing. The sections were immersed into Nissl dye (catalog no. G1430, Solarbio, China) for 10 min at 56°C and washed mildly with water. Then, the sections were differentiated in 80 and 95% ethyl alcohol and in absolute ethyl alcohol for 5 min separately and cleared in xylene for 10 min. Finally, the sections were examined *via* a microscope.

### Immunohistochemistry Staining

The immunohistochemistry staining protocol is identical to the immunofluorescence staining protocol before secondary antibody incubation and differs with respect to immersing the sections in H_2_O_2_. The sections were rinsed in PBS buffer and then incubated in 3% H_2_O_2_ solution for 20 min to remove endogenous peroxidase. Next, the slides were blocked with 5% BSA in 0.01% Triton X-100 in PBS for 1 h at 37°C and then incubated with the following antibodies overnight at 4°C: proBDNF antibody (catalog no. ANT006, 1:1,000, Alomone lab) and NeuN antibody (1:1,000, catalog no. ab104224, Abcam). To test the efficiency of humanized mAb-proB, the same protocol was performed as mentioned above except without the primary antibody. The sections were incubated with biotinylated goat anti-mouse immunoglobulin secondary antibody (1:1,000, ab6788, Abcam) or sheep anti-human IgG H&L (1:1,000, ab6869, Abcam) for 1 h at 37°C. The slides were then washed and incubated in an ABC universal plus kit (catalog no. PK-8200, Vector Laboratories, United States). Finally, 3,3′-diaminobenzidine staining of the sections was performed according to the ABC universal plus kit protocol. Images of the sections were obtained using an optical microscope (Nikon, Japan).

### Reverse Transcription and Quantitative Real-Time PCR

Total RNA extraction from the hippocampus was performed as previously described (Wang et al., 2019; Yu et al., 2020). Briefly, cDNA was obtained using a reverse transcription kit (catalog no. 4368814, Thermo Fisher Scientific, United States). RT-qPCR was carried out with SYBR Green (Bio-Rad) on a CFX96 Touch^TM^ Deep Well Real-Time PCR Detection system (Bio-Rad, Hercules, CA, United States). The primer sequences were as follows: 5′-GGGTGTGAACCACGAGAAAT-3′ and 5′-ACAGTCTTCTGGGTGGCAGT-3′ (*GAPDH*); 5′-AGTGGAGAGTGCTGCAAAGC-3′ and 5′-GTCAGAGAA CGTAACACTGTCCA-3′ (*p75*^*NTR*^); 5′-ATTAGGGAGTGGGT CACAGC-3′ and 5′-GATTGGGTAGTTCGGCATTG-3′ (*BDNF*); 5′-GAAAATGGCCTGTGGGTGTC-3′ and 5′-ACCAAGATC AGCTTTGCAGG-3′ (*sortilin*); 5′-CGCAAACGGCAGGAGA AAGA-3′ and 5′-TGCGCACCTCAGGGCTATTT-3′ (*Trkb*).

### Statistical Analysis

All the experiments were independently performed in triplicate. Data are expressed as mean ± SEM. An unpaired two-tailed Student’s *t*-test, one-way analysis of variance, or two-way analysis of variance was used for statistical analysis. A *p*-value < 0.05 was considered to be statistically significant. The statistical analysis was performed using GraphPad Prism 7.0 (San Diego, CA, United States).

## Results

### Intraperitoneal Injection of LPS Impaired Learning and Memory in Mice

**FIGURE 1:**
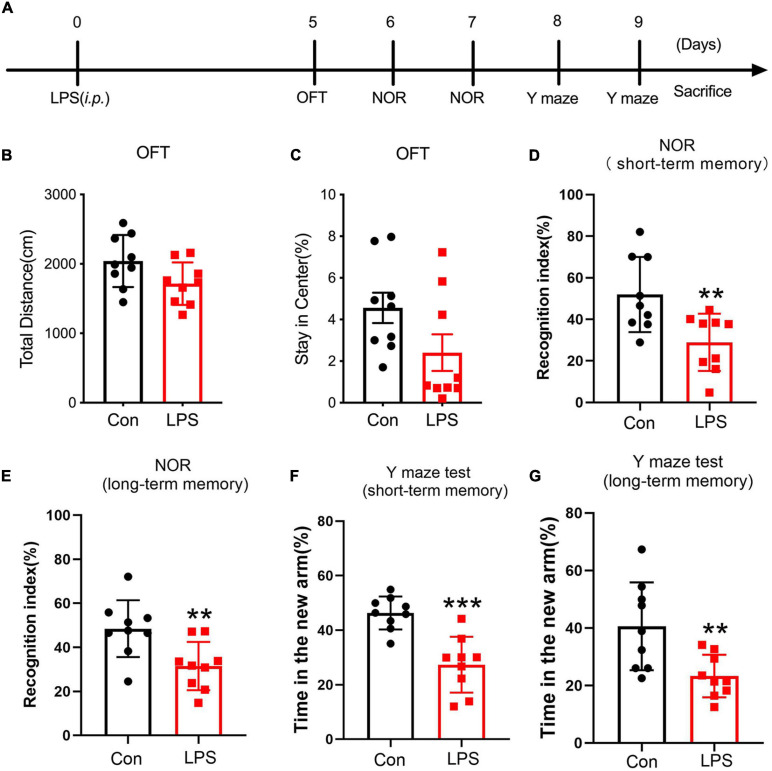
Behavioral tests after the establishment of the sepsis-associated encephalopathy mouse model. **(A)** Timeline of behavioral tests after administration of lipopolysaccharide. **(B,C)** The total distance and the time spent in the center field of the open field test (*n* = 9/group). **(D,E)** The recognition index of short-term or long-term memory tasks in the novel object recognition test (*n* = 9/group). **(F,G)** The percentage of time mice stay in the novel arm in the short-term or long-term memory task in the Y maze test (*n* = 9/group). Data are expressed as mean ± SEM. **p < 0.01, ***p < 0.001.

The behavior routines are shown in Figure 1A for the analysis of the SAE mice model. First, we measured the locomotor activity and emotional behavior using the open field test (OFT) experiment. In the OFT, compared with the control group, there was no significant difference in the locomotor performance of the LPS group on the 5th day after LPS injection (unpaired *t*-test; *t*(16) = 2.012, *p* = 0.0614, Figure 1B) and in the time spent in the center square on the 6th day after LPS injection (unpaired *t*-test; *t*(16) = 1.884, *p* = 0.0779, Figure 1C).

To further assess the influence of LPS on learning and memory in mice, NOR and Y maze tests were conducted (Lin et al., 2018; van der Kooij et al., 2018). Using the NOR and Y maze tests, we found that the mice displayed decreased short-term memory (inter-trial interval: 30 min) (unpaired *t*-test; *t*(16) = 3.035, *p* = 0.0079, Figure 1D; *t*(16) = 4.793, *p* = 0.0002, Figure 1F) and long-term memory (inter-trail interval: 1 day; unpaired *t*-test; *t*(16) = 3.008, *p* = 0.0083, Figure 1E; *t*(16) = 3.059, *p* = 0.0075, Figure 1G) after LPS injection. These data together suggest that i.p. LPS treatment damages the learning and memory functions of mice.

### LPS Injection Altered the Expression of proBDNF, BDNF, and Its Receptors in Mice

**FIGURE 2:**
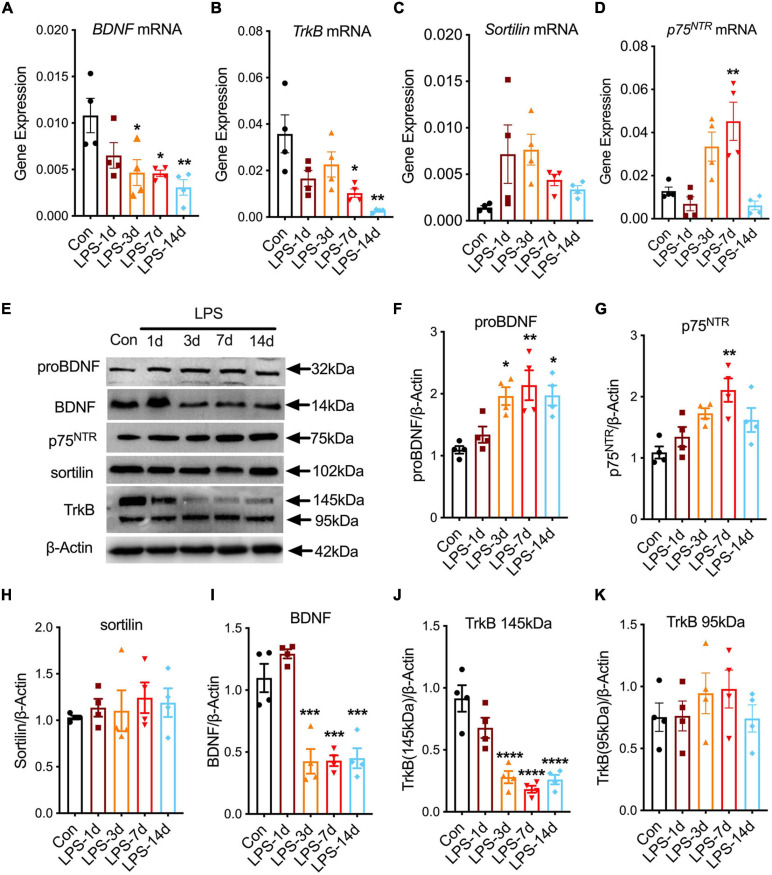
The expression pattern of proBDNF, BDNF, and their receptors in sepsis-associated encephalopathy mice. **(A–D)** The expression level of *BDNF, TrkB, sortilin, and p75^*NTR*^* at different time points after the lipopolysaccharide injection (*n* = 4/group). **(E–K)** Immunoblot analysis of different time points and the quantitative results of the expression of proBDNF, p75^*NTR*^, sortilin, BDNF, and TrkB (*n* = 4/group). Data are expressed as mean ± SEM. *p < 0.05, **p < 0.01, ***p < 0.001, and ****p < 0.0001.

We first examined the expression level of *BDNF*,*TrkB*, *sortilin*, and *p75*^*NTR*^ in the hippocampus of mice on the first, third, seventh, and 14th day after LPS injection (Figures 2A–D). Compared with the control group, the *p75*^*NTR*^ transcripts (Figure 2D) increased on the 7th day after LPS administration (one-way ANOVA, Tukey’s multiple-comparisons test; *F*(4, 15) = 6.093, *p* = 0.0048), the *BDNF* transcripts (Figure 2A) decreased on the third day (one-way ANOVA, Tukey’s multiple-comparisons test; *F*(4, 15) = 4.838, *p* = 0.0267), seventh day (one-way ANOVA, Tukey’s multiple-comparisons test; *F*(4, 15) = 4.901, *p* = 0.0245), and 14th day (one-way ANOVA, Tukey’s multiple-comparisons test; *F*(4, 15) = 6.087, *p* = 0.0048) after LPS administration, and the *TrkB* transcripts (Figure 2B) decreased on the seventh day (one-way ANOVA, Tukey’s multiple-comparisons test; *F*(4, 15) = 5.406, *p* = 0.0123) and 14th day (one-way ANOVA, Tukey’s multiple-comparisons test; *F*(4, 15) = 7.015, *p* = 0.0014) after LPS administration, while the *sortilin* transcripts (Figure 2C) showed no significant change (one-way ANOVA, Tukey’s multiple-comparisons test; *F*(4, 15) = 2.580, *p* > 0.05).

Next, we evaluated the protein levels of BDNF, TrkB, sortilin, and p75^*NTR*^ in the hippocampus of LPS-treated mice (Figures 2E–K). The results showed that, compared with the control group, the protein levels of proBDNF and its high-affinity receptor p75^*NTR*^ increased on day 3 (one-way ANOVA, Tukey’s multiple-comparisons test; *F*(4, 15) = 5.466, *p* = 0.0113 for proBDNF), day 7 (one-way ANOVA, Tukey’s multiple-comparisons test; *F*(4, 15) = 6.569, *p* = 0.0025 for proBDNF; *F*(4, 15) = 6.624, *p* = 0.023 for p75^*NTR*^), and day 14 (one-way ANOVA, Tukey’s multiple-comparisons test; *F*(4, 15) = 5.529, *p* = 0.0104 for proBDNF) after LPS administration, while sortilin showed no significant differences (one-way ANOVA, Tukey’s multiple-comparisons test; *F*(4, 15) = 0.3041, *p* > 0.05; Figures 2E–H). In contrast, the protein levels of mBDNF and its receptor TrkB (145 kDa) decreased on the third day (one-way ANOVA, Tukey’s multiple-comparisons test; *F*(4, 15) = 8.324, *p* = 0.0002 for BDNF; *F*(4, 15) = 9.392, *p* < 0.0001 for TrkB), seventh day (one-way ANOVA, Tukey’s multiple-comparisons test; *F*(4, 15) = 8.262, *p* = 0.0003 for BDNF; *F*(4, 15) = 10.83, *p* < 0.0001 for TrkB), and 14th day (one-way ANOVA, Tukey’s multiple-comparisons test; *F*(4, 15) = 8.014, *p* = 0.0004 for BDNF; *F*(4, 15) = 9.688, *p* < 0.0001 for TrkB) after LPS administration. However, the change in TrkB (95 kDa) levels in SAE mice was not statistically significant (Figures 2E,K). Thus, these results indicate that proBDNF and p75^*NTR*^ are upregulated and BDNF and TrkB (145 kDa) are downregulated following LPS administration.

### proBDNF and Its Receptor p75^*NTR*^ Are Expressed Mainly in Hippocampal Neurons

**FIGURE 3:**
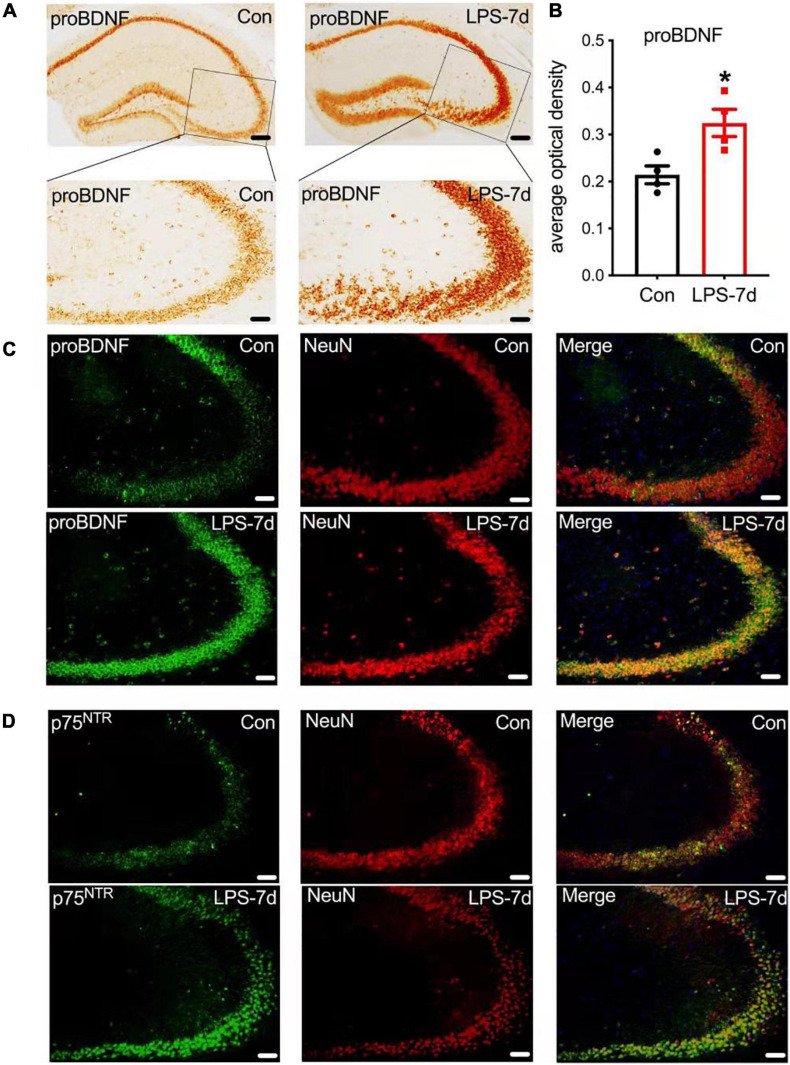
proBDNF and its receptor p75^*NTR*^ are mainly expressed in hippocampal neurons. **(A,B)** proBDNF accumulated in hippocampal neurons (upper scale bar = 100 μm, lower scale bar = 50 μm, *n* = 4/group). **(C,D)** proBDNF and its receptor p75^*NTR*^ mainly co-existed in the neuron (scale bar = 50 μm, *n* = 4/group). Data are expressed as mean ± SEM. *p < 0.05.

Immunohistochemistry staining demonstrated that proBDNF was highly expressed (unpaired *t*-test; *t*(6) = 3.197, *p* = 0.0187) in the hippocampus on the seventh day after LPS injection (Figures 3A,B). To further confirm the distribution of proBDNF and p75^*NTR*^ in hippocampal cells, we applied dual-color immunofluorescence staining of hippocampus tissue sections from SAE mice (Figures 3C,D). The representative tissue stains showed that both proBDNF and p75^*NTR*^ are expressed mainly in neurons in the hippocampus CA3 region (Figures 3C,D). Taken together, the above-mentioned results indicate that upregulated proBDNF may bind p75^*NTR*^ to affect learning and cognition by regulating the neuron function in SAE mice.

### Intrahippocampal Microinjection of the Anti-proBDNF Neutralizing Antibody Improved SAE-Induced Cognitive Dysfunction

**FIGURE 4:**
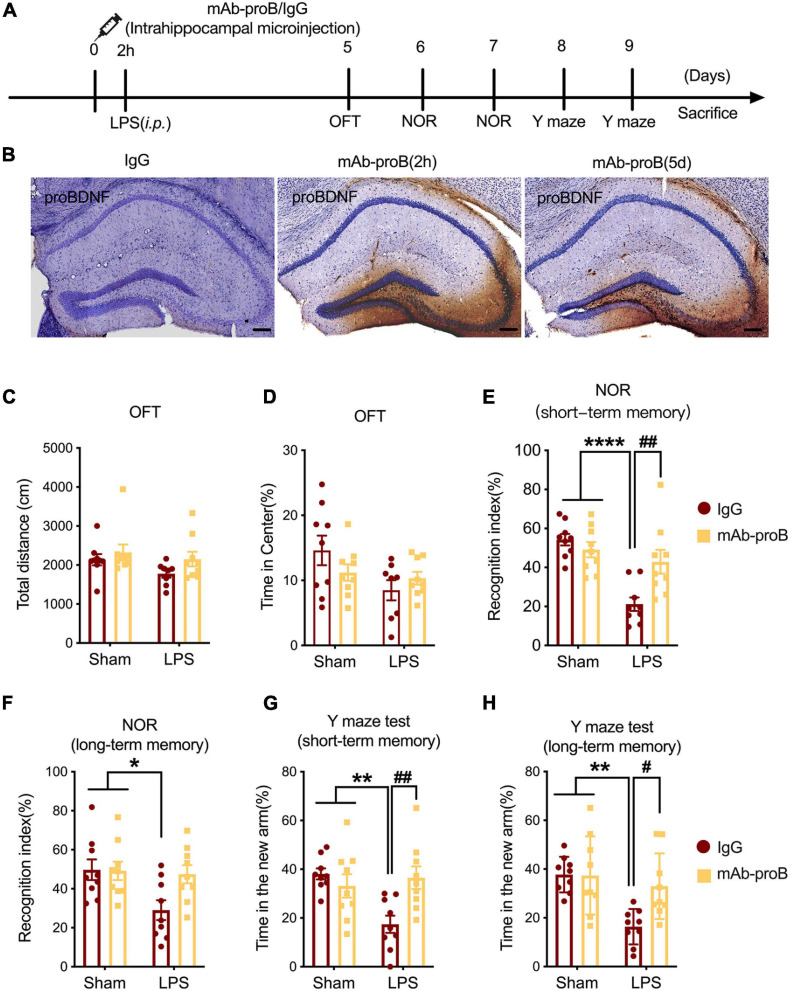
Intrahippocampal microinjection of mAb-proB improved the sepsis-associated encephalopathy-induced cognitive impairment. **(A)** The behavioral intervention flowchart of the experiment. **(B)** The efficiency of neutralizing mAb-proB in hippocampus tissue. Scale bar = 100 μm. **(C,D)** The total traveled distance and percentage of time mice spent in the center of the field *via* open field test (*n* = 9/group). **(E,F)** The recognition index of short/long-term memory tasks of the novel object recognition test (*n* = 9/group). **(G,H)** The percentage of the time that mice stay in the novel arm of the short/long-term memory tasks in the Y maze test (*n* = 9/group). Data are expressed as mean ± SEM. *p < 0.05, **p < 0.01, ****p < 0.0001 vs. sham group; ^#^*p* < 0.05, ^##^*p* < 0.01, LPS + IgG vs. LPS + mAb-proB.

To confirm the role of proBDNF in the learning and memory behavior of LPS-induced SAE mice model, we administered the anti-proBDNF neutralizing antibody (mAb-proB) *via* intrahippocampal microinjection. At 2 h before the i.p. injection of normal saline or LPS, the mice underwent intrahippocampal microinjection with 1 μg of mAb-proB or an equal volume of control IgG. The mice treated with normal saline plus IgG or mAb-proB were defined as the Sham + IgG group and Sham + mAb-proB group, respectively. Behavioral tests in the normal saline group were performed from day 5 to 9 after injection to evaluate the influence of mAb-proB on the cognitive behaviors of four different groups of mice (Sham + IgG, Sham + mAb-proB, LPS + IgG, and LPS + mAb-proB; Figure 4A).

We first measured the efficiency of mAb-proB by immunohistochemistry staining under the intrahippocampal microinjection. To complete this test, we added secondary antibody to test the neutralizing efficiency of mAb-proB, and IgG was used as a negative control. The representative image shows that mAb-proB was still present from 2 h to 5 days after stereotaxic injection, before we performed the behavioral evaluation (Figure 4B).

The locomotive activity and anxiety-like behaviors of the mice were evaluated on the fifth day after mAb-proB stereotaxic injection. The analysis found no significant differences among the four cohorts in the total distance and percentage of time spent in the center square in the OFT experiment (two-way ANOVA, Tukey’s multiple-comparisons test; *F*(1, 31) = 0.3238, *p* > 0.05 for total distances Figure 4C; *F*(1, 31) = 2.719, *p* > 0.05 for percentage of time in center; Figure 4D). Compared with mice in the Sham group, LPS induced short/long-term memory dysfunction in the LPS + IgG group mice (two-way ANOVA, Tukey’s multiple-comparisons test; *F*(1, 32) = 7.711, *p* < 0.0001, for NOR short-term memory Figure 4E; *F*(1, 32) = 6.996, *p* = 0.0283, for NOR long-term memory; Figure 4F; *F*(1, 32) = 5.562, *p* = 0.0041, for Y maze test short-term memory; Figure 4G; two-way ANOVA, *F*(1, 32) = 5.485, *p* = 0.0026, for Y maze test long-term memory; Figure 4H). While the cognitive index of the NOR short-term memory tasks in the LPS + mAb-proB group mice was noticeably higher than that of the LPS + IgG group (two-way ANOVA, Tukey’s multiple-comparisons test; *F*(1, 32) = 5.063, *p* = 0.0059, Figure 4E), there was no significant difference in the improvement of long-term memory between the LPS + IgG and LPS + mAb-proB groups (two-way ANOVA, Tukey’s multiple-comparisons test; *F*(1, 32) = 3.719, *p* > 0.05, Figure 4F). Additionally, the percentage of time spent in the novel arm of the short/long-term memory tasks of the Y maze test (two-way ANOVA, Tukey’s multiple comparisons test; *F*(1, 32) = 4.856, *p* = 0.0086, for short-term memory; Figure 4G; *F*(1, 32) = 4.265, *p* = 0.0244, for long-term memory; Figure 4H) was noticeably higher in the LPS group mice following mAb-proB treatment.

### Systemic Delivery of mAb-proB Partially Reversed the SAE-Induced Cognitive Impairment

**FIGURE 5:**
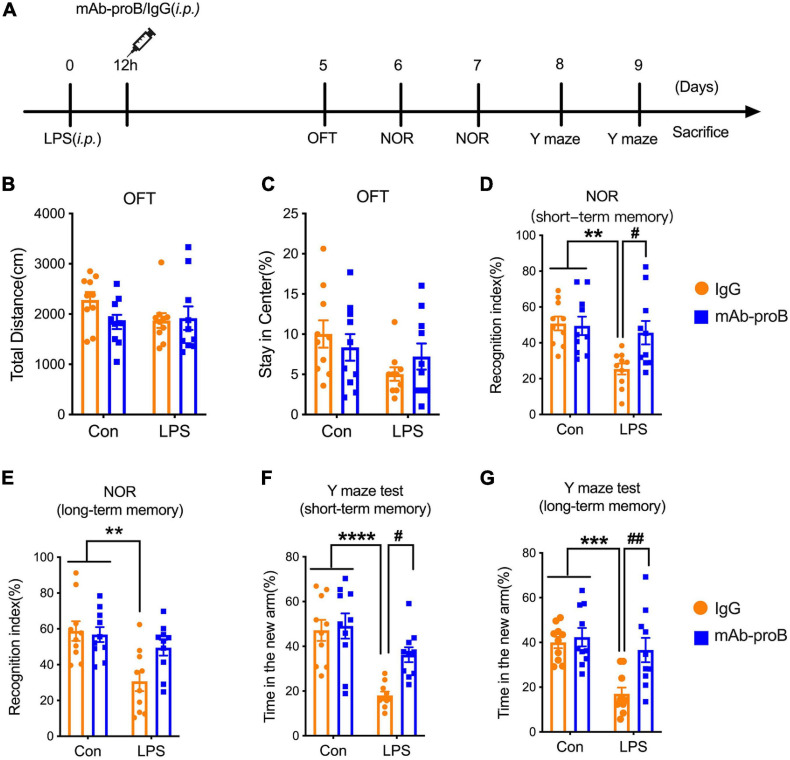
Systemic delivery of neutralizing mAb-proB partially reversed the sepsis-associated encephalopathy-induced cognitive impairment. **(A)** The behavior schedule of the experiment. **(B,C)** The total distance traveled and percentage of time during which mice stay in the center of the field (*n* = 9–10/group). **(D,E)** The recognition index of the short/long-term memory tasks of the novel object recognition test (*n* = 10/group). **(F,G)** The percentage of time that mice stay in the novel arm of the short/long-term memory tasks in the Y maze test (*n* = 10/group). Data are expressed as mean ± SEM. **p < 0.01, ***p < 0.001, ****p < 0.0001 vs. control; ^#^*p* < 0.05, ^##^*p* < 0.01, LPS + IgG vs. LPS + mAb-proB.

While we have demonstrated that an intracerebral injection of mAb-proB can improve SAE-induced cognitive impairment, intracerebral injection is an unlikely delivery method in a clinical setting. Thus, it is important to optimize the delivery of mAb-proB with respect to feasibility and efficiency in our experimental applications. To this effect, we delivered the mAb-proB (100 μg) or the same concentration of mouse IgG *via* i.p. injection 12 h after LPS administration and performed the behavioral tests from day 5 to 9 after LPS administration (Figure 5A).

The OFT results showed that the total distance traveled and the percentage of time spent in the center field was similar between the four groups (two-way ANOVA, Tukey’s multiple-comparisons test; *F*(1, 36) = 1.956, *p* > 0.05 for total distances; Figure 5B; *F*(1, 36) = 1.656, *p* > 0.05 for percentage of time in center; Figure 5C). From the NOR and Y maze tests, we found that LPS administration decreased the short/long-term memory in the SAE mice group (two-way ANOVA, Tukey’s multiple-comparisons test; *F*(1, 36) = 5.233, *p* = 0.0038, for ORT short-term memory; Figure 5D; *F*(1, 36) = 5.673, *p* = 0.0016, for ORT long-term memory; Figure 5E; *F*(1, 36) = 7.075, *p* < 0.0001, for Y maze test short-term memory; Figure 5F; *F*(1, 36) = 5.905, *p* = 0.0010, for Y maze test long-term memory; Figure 5G). Compared with the LPS + IgG group, we found that mAb-proB treatment noticeably rescued the short-term memory impairment of SAE mice (two-way ANOVA, Tukey’s multiple-comparisons test; *F*(1, 36) = 4.173, *p* = 0.0272, Figure 5D) but had no effect on the long-term memory function in the NOR experiment (two-way ANOVA, Tukey’s multiple-comparisons test; *F*(1, 36) = 3.791, *p* > 0.05, Figure 5E). The results of the Y maze test showed that the mAb-proB treatment increased the time that the mice stayed in the novel arm not only with regards to short-term memory (two-way ANOVA, Tukey’s multiple-comparisons test; *F*(1, 36) = 4.426, *p* = 0.0174, Figure 5F) but also with long-term memory (two-way ANOVA, Tukey’s multiple-comparisons test; *F*(1, 36) = 5.030, *p* = 0.0057, Figure 5G).

### MAb-proB Enhanced the Expression of NeuN and Synapse-Associated Proteins

**FIGURE 6:**
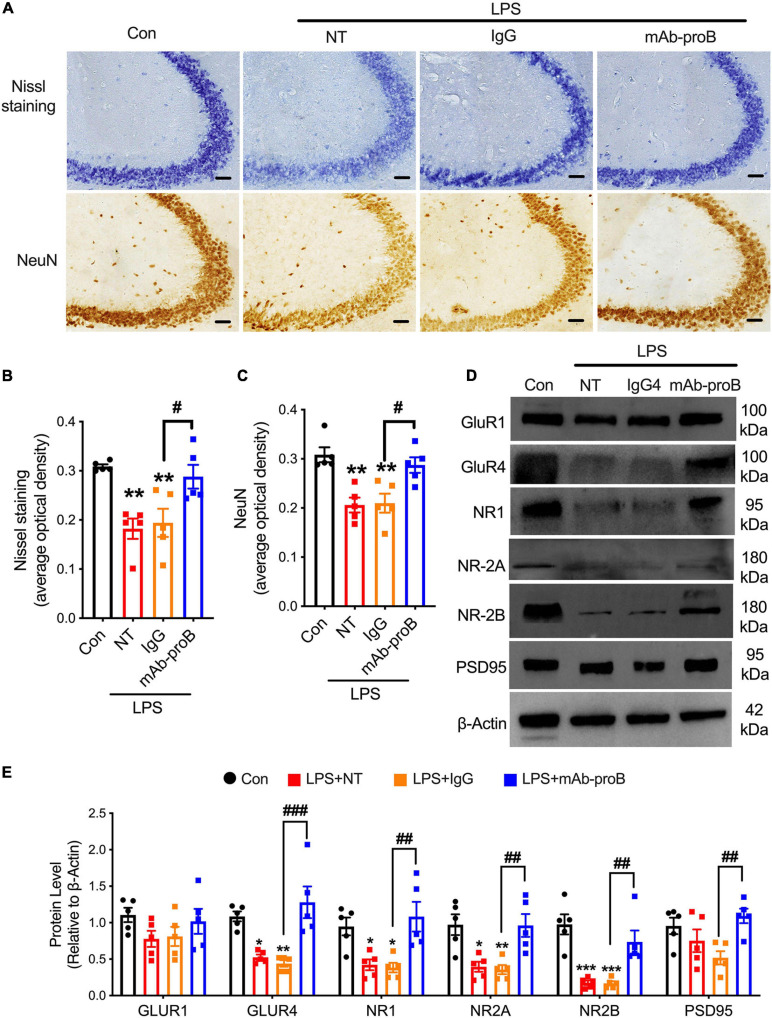
MAb-proB upregulated the expression of neuronal cells and synapse-associated proteins in sepsis-associated encephalopathy mice. **(A–C)** Representative images and quantitation of Nissl bodies and NeuN-positive neuronal cells (scale bar = 50 μm, *n* = 4/group). **(D,E)** Immunoblot analysis of GluR1, GluR4, NR1, NR-2A, NR-2B, and PTSD95 (*n* = 5/group). Data are expressed as mean ± SEM. *p < 0.05, **p < 0.01, ***p < 0.001 vs. control; ^#^*p* < 0.05, ^##^*p* < 0.01, ^###^*p* < 0.001, LPS + IgG vs. LPS + mAb-proB.

To investigate the mechanism of systemic mAb-proB delivery on enhancing cognitive function in SAE mice, we assessed the levels of synapse-associated proteins, Nissl bodies, and NeuN-positive neuronal cells (Figure 6). Compared with the control group, the systemic administration of LPS resulted in the decreased expression of Nissl bodies and NeuN-positive neuronal cells (one-way ANOVA, Tukey’s multiple-comparisons test; *F*(3, 16) = 5.916, *p* = 0.0035, for Nissl staining; Figures 6A,B; *F*(3, 16) = 6.228, *p* = 0.0023, for NeuN staining; Figures 6A–C), while mAb-proB treatment can improve the expression of Nissl bodies (one-way ANOVA, Tukey’s multiple-comparisons test; *F*(3, 16) = 4.385, *p* = 0.0314, Figure 6A) and NeuN (one-way ANOVA, Tukey’s multiple-comparisons test; *F*(3, 16) = 4.725, *p* = 0.0195, Figures 6A–C) in the hippocampus of SAE mice.

Next, we assessed the levels of synapse-associated proteins at 7 days after mAb-proB treatment in the SAE mice. The immunoblot analysis showed that the expression of GluR4 (one-way ANOVA, Tukey’s multiple-comparisons test; *F*(3, 16) = 4.761, *p* = 0.0185), NR1 (one-way ANOVA, Tukey’s multiple-comparisons test; *F*(3, 16) = 4.089, *p* = 0.0472), NR2A (one-way ANOVA, Tukey’s multiple-comparisons test; *F*(3, 16) = 4.966, *p* = 0.0138), and NR2B (one-way ANOVA, Tukey’s multiple-comparisons test; *F*(3, 16) = 7.498, *p* = 0.0004) decreased remarkably in the hippocampus after LPS injection (Figures 6D,E). Compared with the LPS + IgG group, the systemic delivery of mAb-proB resulted in an increase in the hippocampal protein levels of GluR4 (one-way ANOVA, Tukey’s multiple-comparisons test; *F*(3, 16) = 7.142, *p* = 0.0006), NR1 (one-way ANOVA, Tukey’s multiple-comparisons test; *F*(3, 16) = 5.426, *p* = 0.0071), NR2A (one-way ANOVA, Tukey’s multiple-comparisons test; *F*(3, 16) = 5.225, *p* = 0.0095), NR2B (one-way ANOVA, Tukey’s multiple-comparisons test; *F*(3, 16) = 5.324, *p* = 0.0244), and PSD95 (one-way ANOVA, Tukey’s multiple-comparisons test; *F*(3, 16) = 5.282, *p* = 0.0248) (Figures 6D,E).

To understand the effects of mAb-proB treatment on the expression of BDNF, TrkB, and sortilin in the hippocampus, BDNF, TrkB, and sortilin expression was analyzed by RT-qPCR. Compared with the IgG group, the mAb-proB treatment of SAE mice had no effect on the gene expression of BDNF, TrkB, and sortilin (Supplementary Figure 1).

Taken together, these results suggest that mAb-proB may improve learning and memory dysfunction through enhancing the expression of NeuN and synapse-associated proteins in the hippocampus of SAE mice.

## Discussion

This study illustrated the role of proBDNF in the regulation of learning and memory dysfunction in SAE mice. We found that SAE induced cognitive impairment in mice and was associated with an increased expression of proBDNF and p75^*NTR*^ and a decreased expression of BDNF and TrkB in the hippocampus. Furthermore, intrahippocampal microinjection or systemic delivery of neutralizing mAb-proB attenuated the cognitive impairment of SAE mice. The mechanism by which mAb-proB (i.p.) ameliorates SAE-induced cognitive impairment may be related to the enhanced expression of NeuN and synapse-associated proteins in the hippocampus.

Studies reported SAE as an early indication of sepsis; a critical clinical sign for SAE is cognitive dysfunction (Gofton and Young, 2012). As a major component of Gram-negative bacteria cell wall (Tang et al., 2007), LPS was reported to induce neuron toxicity (Zhu et al., 2015), brain dysfunction, and memory disorders (Choi et al., 2017; Han et al., 2018). LPS injection is a widely used and easily replicated experimental model of SAE. This model induces an overwhelming activation of the innate immune system, which has a number of similarities to SAE (Fink, 2014; Liao et al., 2020). Hence, we performed ORT and Y maze tests to evaluate the short- and long-term learning and memory behavior in this LPS-based mice model. In this study, after 5 days of LPS administration, serious short- and long-term cognitive impairment was observed in SAE mice in ORT and Y maze tests, while no anxiety-like behaviors was found *via* the OFT experiment (Figure 1). Thus, systemic LPS administration led to cognitive impairment in the SAE mouse model.

The hippocampus is the key region for learning and memory (Zeidman and Maguire, 2016; Fares et al., 2019; Zeidman and Maguire, 2016; Fares et al., 2019) which is frequently and easily affected by inflammation (Zong et al., 2019). However, the mechanism of SAE-induced cognitive impairment has not been fully elucidated. Studies indicated that the downregulation of neurotrophic factors, especially BDNF, plays an important role in cognitive impairment and neurodegeneration (Fleitas et al., 2018; Caffino et al., 2020). BDNF is one of the neurotrophic factors that are expressed widely in the brain, regulating neuronal survival, synaptic transmission, synaptic plasticity, and learning and memory (Song et al., 2017). Della Giustina et al. (2020) found that BDNF was remarkably decreased on day 10 after sepsis induction in rats, and those animals displayed a severe cognitive impairment. Pang et al. (2004) reported that BDNF supported neuronal survival, long-term potentiation, and learning and memory. As the precursor of BDNF, proBDNF, other than producing BDNF, also binds to its receptors to perform multiple biological functions in the CNS (Luo et al., 2016). Upregulated proBDNF-induced apoptosis suppressed hippocampal neuron remodeling (Montroull et al., 2019), synaptic transmission (Yang et al., 2009), and synaptic plasticity (Yang et al., 2014). Increased proBDNF and its receptor p75^*NTR*^ were detected in SAE mice (Thomas et al., 2016; Ji et al., 2018). In our study, we identified that proBDNF and p75^*NTR*^ increased steadily upon LPS injection and reached a peak on day 7 after LPS administration. Concurrently, the expression of BDNF and its receptor TrkB decreased in the hippocampus of SAE mice (Figure 2). These results suggest that the imbalance between proBDNF and BDNF might be associated with cognitive impairment in the SAE model. Additionally, immunofluorescence staining revealed that proBDNF and p75^*NTR*^ are mainly expressed in neurons (Figure 3). Thus, elevated proBDNF may bind to p75NTR and affect learning and cognition by regulating the neuron function in SAE mice.

Previous studies showed that mAb-proBDNF treatment leads to the growth of synapses and the improvement of emotional disorders in animal models (Sun et al., 2012; Zhong et al., 2019). An et al. (2018) reported that a hippocampus injection of mAb-proBDNF promotes the location learning strategy of rats. Additionally, an intraperitoneal injection of mAb-proBDNF remarkedly alleviated emotional dysfunction (Bai et al., 2016; Zhong et al., 2019). We also observed that an intraperitoneal injection of mAb-proBDNF highly alleviated the fear conditioning memory in SAE mice, which was accomplished by perturbing the peripheral proinflammatory response (Luo et al., 2020). In our latest study, we confirmed that mAb-proBDNF treatment can attenuate multiple sclerosis in a mouse model and inhibit the proinflammatory response in the spinal cord and spleen of diseased mice (Hu et al., 2021). In the current study, our results showed that both intrahippocampal microinjection (Figure 4) and the systemic delivery (Figure 5) of mAb-proBDNF improved the cognitive impairment in SAE mice, this may be due to the inhibition of the inflammatory response of the CNS and/or peripheral system.

Interestingly, our results also found that mAb-proBDNF is not helping to improve long-term memory impairment in the NOR test. The hippocampus was known to be involved in object memorization and long-term object recognition (Reger et al., 2009; Antunes and Biala, 2012). Studies have shown that the downregulation of BDNF impaired long-term, but not short-term, memory recognition (Alonso et al., 2002; Lee et al., 2004; Seoane et al., 2011). BDNF was shown to regulate object recognition memory reconsolidation through the induction of long-term potentiation (Radiske et al., 2017; Rossato et al., 2019). In our study, we found that mAb-proBDNF treatment did not influence the expression of BDNF, TrkB, and sortilin in the hippocampus (Supplementary Figure 1). This indicates that downregulated BDNF may impair the long-term memory of novel objects in SAE mice. Notably, it is well established that i.p. LPS (10 mg/kg) can disrupt the integrity of the blood–brain barrier (BBB) in mice (Liu et al., 2020). Taken together, we speculate that mAb-proBDNF may cross the BBB to regulate learning and memory dysfunction directly in the SAE mice.

To further explore the function of systemic mAb-proBDNF treatment in SAE mice, we assessed the expression of NeuN and Nissl bodies in the hippocampus. An i.p. LPS administration resulted in a marked decrease in the expression of Nissl bodies and NeuN, while a systemic mAb-proB treatment enhanced the expression of those proteins (Figure 6). NeuN, a neuron-specific nuclear protein located in the neuronal nucleus, is involved in the regulation of mRNA splicing and played a role in regulating neural cell differentiation and the development of the nervous system (Kim et al., 2009; Li et al., 2015; Duan et al., 2016). The Nissl body was composed of many rough endoplasmic reticula and scattered ribosomes which synthesize the proteins during organelle renewal (Kádár et al., 2009). These results suggested that mAb-proB may regulate the function of hippocampal neuronal cells in LPS-induced SAE mice.

Synapse-associated proteins, such as NMDA receptors and PSD95, play a key role in learning and memory. Previous studies suggested that synaptic protein damage was associated with cognitive impairment (Zhang et al., 2017; Merino-Serrais et al., 2019). The GluR2B levels in the frontal cortex decreased 28 h after LPS treatment (Savignac et al., 2016), and the selectivity of NMDA receptors was also reduced (Zhang et al., 2017). Consistent with previous studies (Liraz-Zaltsman et al., 2016; Zhang et al., 2017; Muhammad et al., 2019), we found that the GluR4, NR1, NR2A, and NR2B levels decreased in hippocampal tissue on day 7 after LPS administration in SAE mice. Interestingly, the expression of GluR4, NR1, NR2A, NR2B, and PSD95 can be rescued after mAb-proB treatment (Figure 6). In summary, these data indicate that the neutralizing proBDNF antibody may improve the learning and memory dysfunction *via* enhancing the function of neuronal cells and the expression of synapse-associated proteins in the hippocampus in a mouse model of sepsis.

The current therapies of SAE disease are, to a large extent, hampered by the inability of drugs to cross the BBB. Thus, targeting the BBB should be incorporated as part of a short- and long-term therapeutic strategy in sepsis patients. The proBDNF antibody acts as a macromolecular substance; under normal circumstances, it is difficult to penetrate the BBB. In our study, we speculated that the peripheral delivery of the proBDNF antibody may partially reach the brain and play a regulatory function under the LPS-induced SAE disease mice model. Nevertheless, the ability and efficiency of the proBDNF antibody to penetrate the BBB should be improved in our future study. Recent studies by Kariolis et al. (2020) and Ullman et al. (2020) provided a feasible method to modify the BBB penetration ability of proBDNF antibodies. Using the Fc fragment BBB transport vehicle platform to design the fusion protein combined with the proBDNF antibody will help to improve the penetration ability of the proBDNF antibody into the brain. This method will provide more possibilities for the proBDNF antibody for the treatment of human CNS diseases, such as SAE. In addition, p75^*NTR*^ is the main high-affinity receptor of proBDNF. Therefore, the development of BBB-permeable small-molecule p75^*NTR*^ signaling modulator also has the potential to treat pathogenetic diseases related to proBDNF.

## Conclusion

In conclusion, the present study reports a regulatory function of proBDNF on hippocampal neuronal cells and its detrimental role in the pathogenesis of SAE. Treatment with mAb-proBDNF may effectively attenuate cognitive impairment and represent a potential therapeutic strategy for treating patients with SAE.

## Data Availability Statement

The original contributions presented in the study are included in the article/Supplementary Material, further inquiries can be directed to the corresponding author/s.

## Ethics Statement

The animal study was reviewed and approved by Animal Care and Use Committee of Central South University.

## Author Contributions

Y-HC, S-FZ, Z-LH, and C-QL contributed to conception and design of the study. S-FZ organized the database. SW performed the statistical analysis. Y-HC wrote the first draft of the manuscript. YL, S-FZ, R-PD, and FL wrote sections of the manuscript. All authors contributed to manuscript revision, read, and approved the submitted version.

## Conflict of Interest

The authors declare that the research was conducted in the absence of any commercial or financial relationships that could be construed as a potential conflict of interest.

## Supplementary Material

Supplementary Figure 1 mAb-proB has no impact on the expression of BDNF, TrkB, and sortilin in the hippocampus of sepsis-associated encephalopathy mice. The expression levels of BDNF **(A)**, TrkB **(B)**, and sortilin **(C)** in the hippocampus tissue were measured by RT-qPCR (*n* = 6/group). Data are expressed as mean ± SEM.

## Abbreviations

SAE: sepsis-associated encephalopathy
BDNF: brain-derived neurotrophic factor
LPS: lipopolysaccharide
TrkB: tropomyosin receptor kinase B
CNS: central nervous system
mBDNF: mature brain-derived neurotrophic factor
OFT: open field test
NOR: novel object recognition test
PVDF: polyvinylidene fluoride
TBST: Tris-buffered saline Tween-20.
