# Supplementary material for: Injection of Anti-proBDNF Attenuates Hippocampal-Dependent Learning and Memory Dysfunction in Mice With Sepsis-Associated Encephalopathy
Source: Front Neurosci. 2021 Jul 20;15:665757. doi: 10.3389/fnins.2021.665757 (PMC8329425; doi:10.3389/fnins.2021.665757)
Supplement: Supplementary Figure 1 — mAb-proB has no impact on the expression of BDNF, TrkB, and sortilin in the hippocampus of sepsis-associated encephalopathy mice. The expression levels of BDNF (A), TrkB (B), and sortilin (C) in the hippocampus tissue were measured by RT-qPCR (n = 6/group). Data are expressed as mean ± SEM. [file Image_1.pdf]

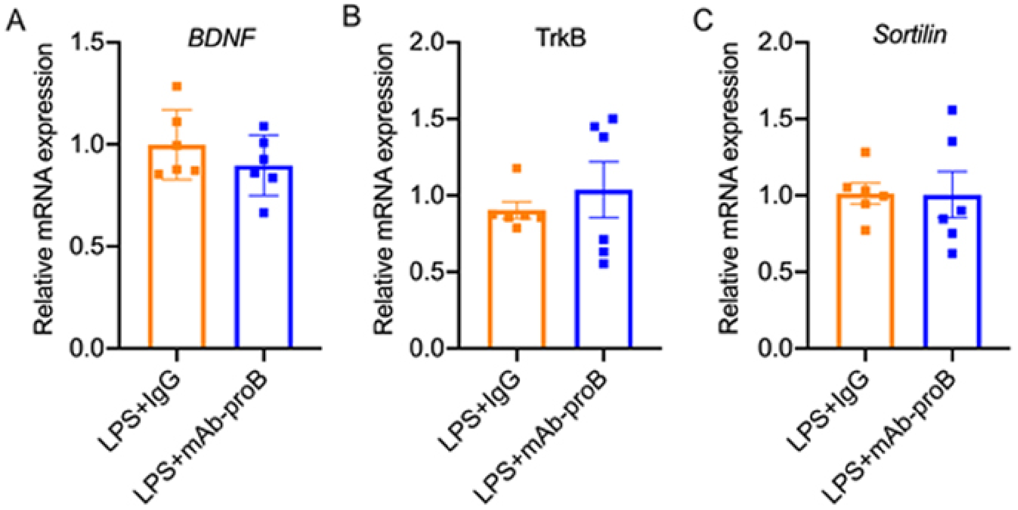

Supplementary Fig. 1. mAb-proB has no impact on the expression of BDNF, TrkB and sortilin in the hippocampus of SAE mice. The expression levels of BDNF (A), TrkB (B) and sortilin (C) in hippocampus tissue were measured by RT-qPCR (n=6/group). Data are expressed as mean ± SEM.
